# Poor removal of tedizolid during continuous hemodiafiltration: experiments using an in vitro continuous hemodiafiltration model

**DOI:** 10.1186/s40780-023-00307-9

**Published:** 2023-11-14

**Authors:** Satoshi Yoshikawa, Shinichi Yoshikawa, Akira Sato, Tsukasa Matsumoto

**Affiliations:** 1 Faculty of Pharmacy, Iryo Sosei University, 5-5-1 Iino, Chuo-Dai, Iwaki, Fukushima 970-8551 Japan; 2 Graduate School of Life Science and Technology, Iryo Sosei University, 5-5-1 Iino, Chuo-Dai, Iwaki, Fukushima 970-8551 Japan

**Keywords:** Tedizolid, Continuous hemodiafiltration, Methicillin-resistant Staphylococcus aureus

## Abstract

**Background:**

Tedizolid is an oxazolidinone anti-MRSA drug with included in the National Health Insurance Drug Price List in 2018. The effect of hemodialysis on tedizolid phosphate concentrations has been reported; pre-dialysis concentrations decreased by 10% compared to post- dialysis concentrations. However, the material of the dialysis membrane remains unknown. In addition, there have been no reports on the effects of continuous hemodiafiltration. In this study, we investigated the effects of continuous hemodiafiltration on tedizolid using two types of dialysis membranes made of different materials.

**Methods:**

The adsorption of tedizolid, linezolid, and vancomycin to two different dialysis membranes was investigated, and the clearance of each drug was calculated by experiments using an in vitro continuous hemodiafiltration model.

**Results:**

The adsorption of tedizolid, linezolid, and vancomycin on the dialysis membranes was examined, and no adsorption was observed. Experimental results from the continuous hemodiafiltration model showed that linezolid and vancomycin concentrations decreased over time: after two hours, the respective decreases were 26.48 ± 7.14% and 28.51 ± 2.32% for polysulfone membranes, respectively. The decrease was 23.57 ± 4.95% and 28.73 ± 5.13% for the polymethylmethacrylate membranes, respectively. These results suggested that linezolid and vancomycin were eliminated by continuous hemodiafiltration. In contrast, tedizolid phosphate and tedizolid concentrations decreased slightly in the polysulfone and polymethylmethacrylate membranes. The decrease in concentrations were 2.10 ± 0.77% and 2.97 ± 0.60% for the polysulfone membranes, respectively. For the polymethylmethacrylate membranes, the decrease in concentration were 2.01 ± 0.88% and 1.73 ± 0.27%, respectively.

**Conclusion:**

These results suggested that tedizolid should not be considered for dose control during continuous hemodiafiltration.

## Introduction

Patients with severe infections or sepsis caused by methicillin-resistant Staphylococcus aureus (MRSA) often develop complications resulting from acute renal dysfunction or changes in other organ functions. In such cases, hemodiafiltration (HDF), particularly continuous hemodiafiltration (CHDF), which has less impact on blood flow, is widely used in intensive care units. In patients using HDF or CHDF, the optimal dose of antimicrobial agents, including anti-MRSA agents, should be determined based on the patient's renal function and the estimated efficiency of drug clearance by the dialysis membrane [1, 2]. When vancomycin (VCM), the most commonly used anti-MRSA agent, is administered to remove mediators, such as inflammatory cytokines, in patients with adequate urine output, its clearance is affected by both renal function and clearance by CHDF, necessitating dose escalation by therapeutic drug monitoring (TDM) [3].

Sivextro® (tedizolid phosphate) is a novel oxazolidinone antimicrobial agent indicated for MRSA infections, which was listed in the National Health Insurance Drug Price List in May 2018. It is similar to the oxazolidinone antimicrobial agent, linezolid (LZD), which binds to the 50S subunit of the ribosome and inhibits the formation of the 70S initiation complex, thereby inhibiting bacterial protein synthesis and growth. Tedizolid phosphate is a prodrug that is converted in vivo into its active form, tedizolid (TZD), which exhibits antimicrobial activity. Regarding the effect of hemodialysis on TZD, the interview form reported a 10% decrease in TZD concentration before dialysis compared with after dialysis. However, the material of the dialysis membrane remains unknown. In addition, there have been no reports on the effects of CHDF. Some drugs are lost in HDF and CHDF by diffusion and ultrafiltration and also by adsorption to the filtration membrane [4–6]. Anti-MRSA agents are an example; the adsorption of these antimicrobials to the dialysis membranes is an important but largely ignored area of research.

The aim of this study was designed to compare the adsorption of tedizolid phosphate and TZD on two types of dialysis membranes with different membrane structures, mainly used in CHDF with other anti-MRSA agents (LZD and VCM) and to investigate the potential for a CHDF-induced decrease in TZD concentration through in vitro experiments.

## Materials and methods

### Reagents

Tedizolid phosphate standard samples were purchased from MedChemExpress (Monmouth Junction, NJ, USA), and TZD was purchased from Nacalai Tesque Corporation (Kyoto, Japan). Sivextro (tedizolid phosphate) and sodium acetate were purchased from MSD Corporation (Tokyo, Japan) and Nacalai Tesque Corporation, respectively. LZD, VCM, acetonitrile, and methanol were purchased from the FUJIFILM Wako Pure Chemical Corporation (Osaka, Japan). Bovine serum albumin (BSA) was added to saline as pseudo-blood and was purchased from Iwai Chemical Corporation. A syringe filter (13 mm cellulose acetate 0.2 μm (AGC Techno Glass Company Limited, Tokyo, Japan) was used for sample solution pretreatment. A saline solution (Otsuka saline solution) was purchased from Otsuka Pharmaceutical Corporation (Tokyo, Japan).

### Comparison of adsorption for dialysis membranes

The adsorption rates of tedizolid phosphate, TZD, LZD, and VCM onto two types of dialysis membranes were investigated (Table 1). The experiments were performed using two types of dialysis membranes: polysulfone (PS) (SNV-1.3, Toray Medical, Tokyo, Japan) and polymethylmethacrylate (PMMA) (CH-1.3, Toray Medical, Tokyo, Japan). First, tedizolid phosphate, TZD, LZD, and VCM were dissolved in 50 ml of saline containing 4% BSA. The drug concentrations used in the experiments were 3.5 μg/ml, 3.5 μg/ml, 20 μg/ml, and 50 μg/ml, referring to the maximum blood concentration (C_max_) of each drug at clinical administration.

**Table 1:**
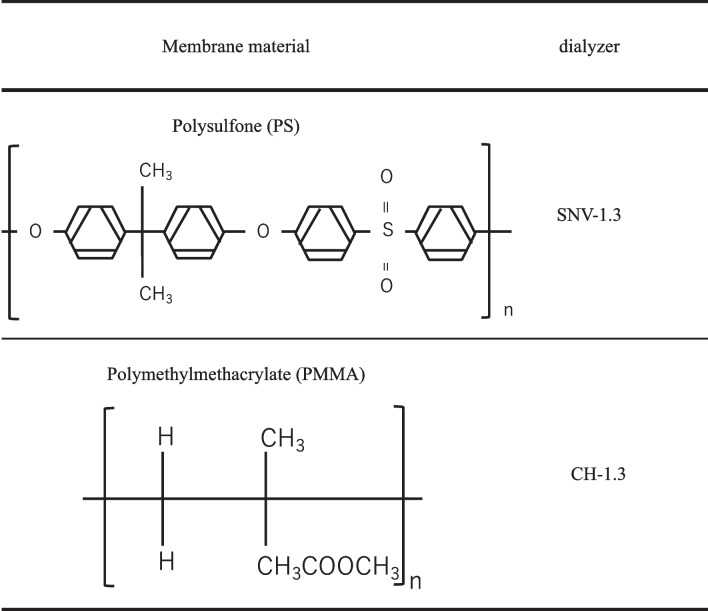
Membrane materials and their molecular structures

The hollow fibers (1 cm wide, 0.1 g) of each dialysis membrane were soaked in solutions of tedizolid phosphate, TZD, LZD, and VCM, stirred for 1 h at room temperature, and collected. To 0.2 ml of this sample, an equal volume of methanol was added (0.2 ml), and the proteins were removed by centrifugation (10,000 g, 25 °C, 30 min). Protein-free samples were assayed by high- performance liquid chromatography (HPLC).

### In vitro CHDF model

**Fig. 1:**
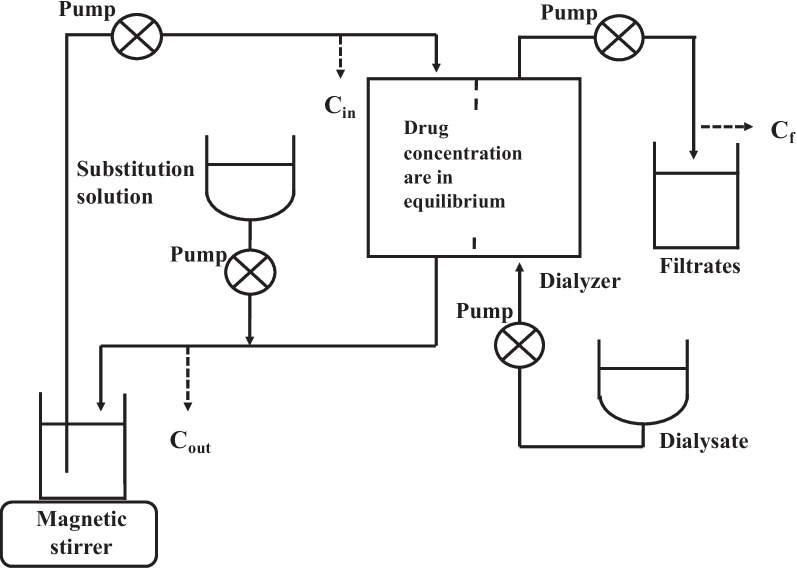
In vitro CHDF model and sampling points. Arrows indicate the flow of solutions, and dotted arrows indicate the sampling points

CHDF was performed using an in vitro model with a blood purification system (KM-8700EX, SANYO Electric Corporation, Japan) (Fig. 1). Saline solution containing 4% BSA was placed in a 1 L beaker and used as pseudo blood. Two types of dialysis membranes with the same composition (PS and PMMA) as those used for the affinity dialysis experiments were used. Drug concentrations were measured using HPLC. The drug concentration used in the experiment was the Cmax of each drug since the experiment will be comparing adsorption rates onto the dialysis membrane. The drug-containing pseudo-blood was circulated through the two types of dialysis membranes (PS and PMMA) and a hemoperfusion blood circuit (Kawasumi Chemical Industries, Tokyo, Japan) at a flow rate (Q_b_) of 100 ml/min. The flow rate of the dialysate (Q_d_) was set to 450 ml/hr, and the flow rates of the substitution solution (Q_s_) and ultrafiltrate (Q_f_) were set to 200 ml/hr. These flow rates were set based on the actual clinical flow rates. The pseudo-blood containing the drug solution was circulated in the circuit to equalize the concentration of the drug solution, and the experiment was then started. Samples were collected from the inlet (C_in_), outlet (C_out_), and filtrate (C_f_) of the dialyzer at 0, 0.5, 1, and 2 h.

### Calculations

The elimination rates of tedizolid phosphate, TZD, LZD, and VCM were calculated as follows:

$$\text{Elimination rate}\ (\mathrm{\%}) = (\mathrm{C}_0-\mathrm{C_n})\,/\mathrm{C}_0 \times 100$$

C_0_: Initial drug concentration

C_n_: Drug concentration at the time of sampling

The clearance of each drug in the in vitro CHDF (CL_CHDF_) was calculated using the following equation:

$$\mathrm{CL}_{\text{CHDF}} =\mathrm{Q}_{\text{b}}\times (\mathrm{C}_{\text{in}}-\mathrm{C}_{\text{out}})/\mathrm{C}_{\text{in}}$$

### HPLC analysis

Analyses of tedizolid phosphate, TZD, LZD, and VCM were performed by HPLC using a Hibar Lichrosorb® RP-18 column (ODS, 5 μm, 4.0 × 120 mm, Kanto Chemical Corporation, Tokyo, Japan). The analyses of tedizolid phosphate and TZD were performed in accordance with previous reports [7–9]. The mobile phase for tedizolid phosphate was a mixture of 19.2 mM sodium acetate buffer (pH 7.4) and 15% acetonitrile. The mobile phase for TZD was a mixture of 19.2 mM sodium acetate buffer (pH 7.4) and 50% MeOH. The flow rate was 1.0 ml/min, and TZD was measured by UV absorbance at 251 nm. The LZD analysis was performed as previously described [10, 11]. The mobile phase was a mixture of 19.2 mM sodium acetate buffer (pH 7.4) and 30% acetonitrile at a flow rate of 1.0 ml/min. The LZD analysis was performed by measuring the UV absorbance at 253 nm. The VCM analysis was performed as previously described [12–14]. The mobile phase was a mixture of 50 mM sodium dihydrogen phosphate buffer (pH 2.5) and 10% acetonitrile at a flow rate of 1.0 ml/min.

### Statistical analysis

Data are expressed as the mean ± SE. One-way analysis of variance (ANOVA) was applied to nonreplicated measurements. Repeated-measures ANOVA followed by the Tukey–Kramer test was applied for repeated or serial determinations. Statistical significance was set at *P* < 0.05.

## Results

### Comparison of adsorption for dialysis membranes

**Fig. 2:**
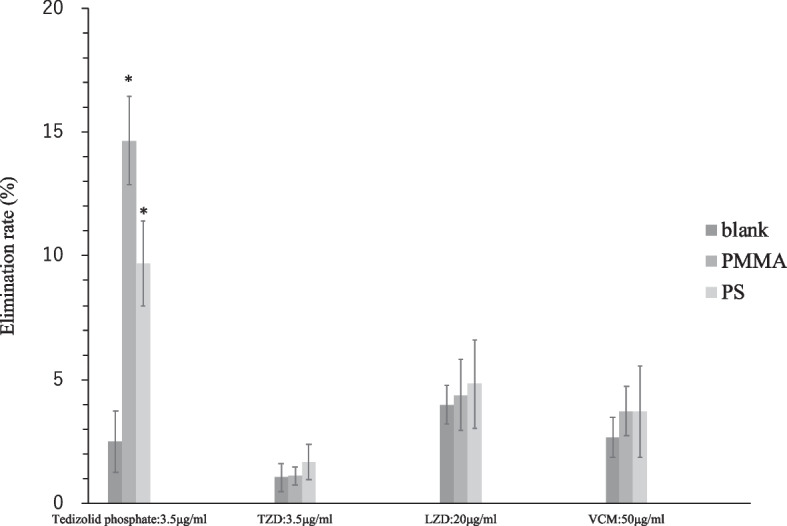
Elimination rates of tedizolid phosphate, TZD, LZD, and VCM on PMMA and PS menbranes. Data are expressed as the mean ± SD (*n* = 4). * Significantly different from the brank control (one-way analysis of variance followed by Tukey–Kramer test, *P* < 0.05). PS = polysulfone, PMMA = polymethylmethacrylate, TZD = tedizolid, LZD = linezolid, VCM = vancomycin

The elimination rate of tedizolid phosphate onto the PS and PMMA membranes after 1 h significantly increased for both membranes compared with that of the control (Fig. 2). However, TZD, LZD, and VCM showed no significant differences between the membranes (Fig. 2).

### Elimination by in vitro CHDF model

**Fig. 3:**
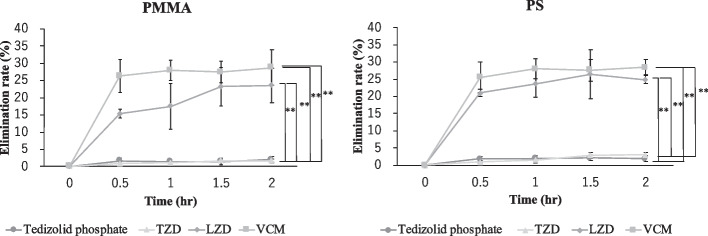
Changes over time in the elimination rates of tedizolid phosphate, TZD, LZD, and VCM. Data are expressed as the mean ± SD (*n* = 4). The vertical axis is the elimination rate, and the horizontal axis is time. Samples were taken over time at 0, 0.5, 1, and 2 h. The elimination rate was calculated from the results obtained. ** Significantly different from the brank control (one-way analysis of variance followed by Tukey–Kramer test, *P* < 0.01)

The elimination rates after 2 h of tedizolid phosphate, TZD, LZD, and VCM were 1.48 ± 0.38% to 2.01 ± 0.88%, 0.88 ± 0.63% to 1.73 ± 0.27%, 15.36 ± 1.23% to 23.57 ± 4.95%, and 26.30 ± 4. 78% to 28.73 ± 5.13% for the PMMA membrane and 1.86 ± 0.76% to 2.10 ± 0.77%, 0.91 ± 0.38% to 2.97 ± 0.60%, 21.01 ± 0.98% to 26.48 ± 7.14%, and 25.56 ± 4.36% to 28.51 ± 2.32% for the PS f membrane (Fig. 3). The elimination rate of tedizolid phosphate, and TZD was significantly lower than that of LZD and VCM (*P* < 0.01). The elimination rates of tedizolid phosphate, TZD, LZD and VCM under BSA-free conditions were 12.31 ± 0.29%, 9.75 ± 1.97%, 25.30 ± 5.28% and 27.39 ± 3.04% for the PMMA membrane and 6.58 ± 0.86%, 9.88 ± 1.40%, 19.82 ± 2.22% and 27.14 ± 2.87% for the PS membrane, respectively.

**Fig. 4:**
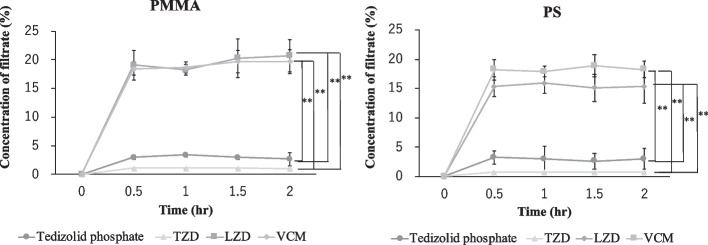
Changes in filtrate concentrations of tedizolid phosphate, TZD, LZD, and VCM in time-course. Data are expressed as the mean ± SD (*n* = 4). The vertical axis is the filtrate concentration, and the horizontal axis is time. Samples were taken over time at 0, 0.5, 1, and 2 h. The adsorption-dependent elimination rate was calculated from the results obtained. ** Significantly different from the brank control (one-way analysis of variance followed by Tukey–Kramer test, *P* < 0.01)

Concentrations after 2 h of tedizolid phosphate, TZD, LZD, and VCM filtrates ranged from 2.68 ± 1.12% to 3.40 ± 0.26%, 1.06 ± 0.01% to 1.09 ± 0.01%, 18.21 ± 0.92% to 20.69 ± 2.74%, and 18.43 ± 1.19% to 19.73 ± 2.08% were detected in the PMMA membrane, 2.59 ± 1.36% to 3.21 ± 1.18%, 0.71 ± 0.01% to 0.72 ± 0.01%, 15.09 ± 2.26% to 15.93 ± 1.75%, and 17.90 ± 0.91% to 18.88 ± 1.88% were detected in the PS membrane (Fig. 4). The filtrate concentrations of tedizolid phosphate, and TZD was significantly lower than that of LZD and VCM (*P* < 0.01).

**Fig. 5:**
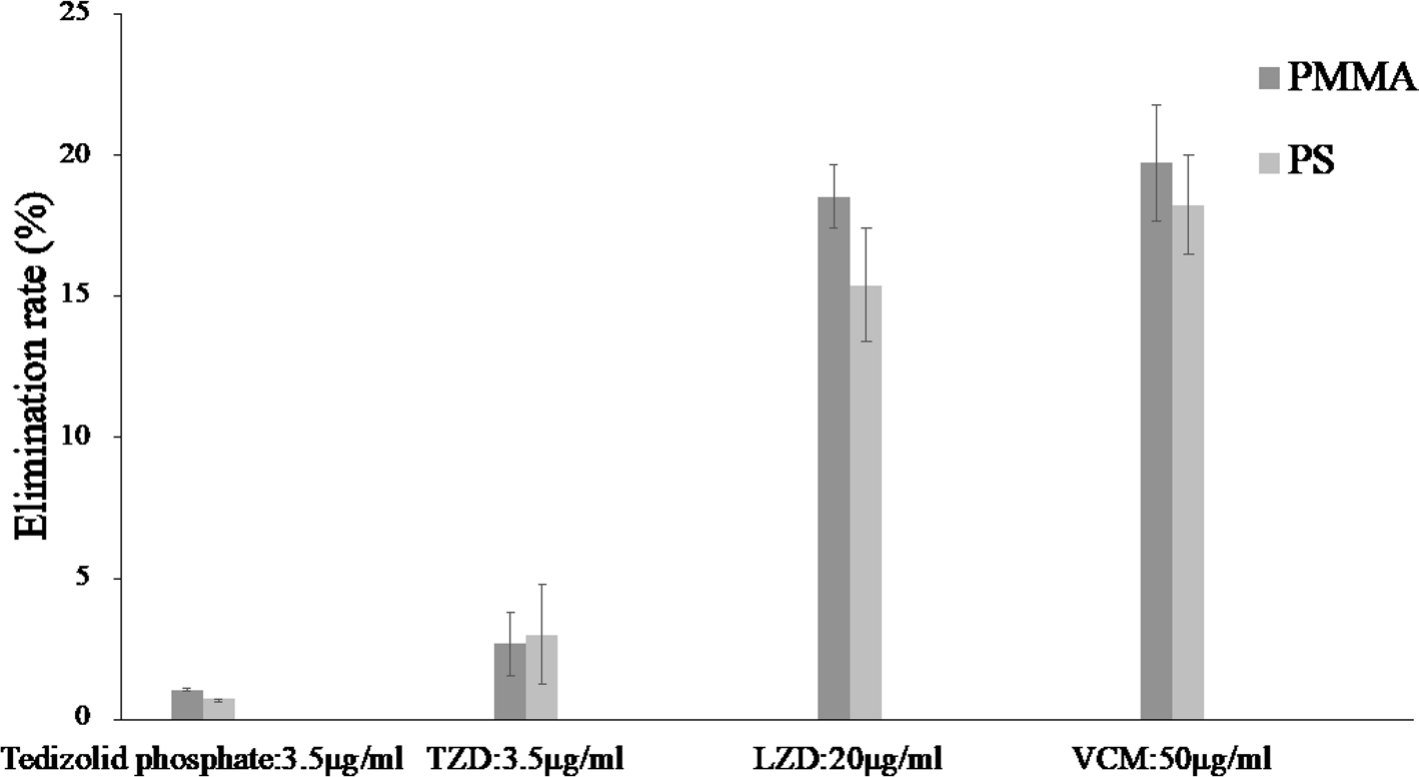
Elimination rate of each drug after 2 h of in vitro CHDF experiments. Data are expressed as the mean ± SD (*n* = 4). The vertical axis is elimination rate, and the horizontal axis is time. Samples were taken over time at 0, 0.5, 1, and 2 h. The elimination rate was calculated from the results obtained

Comparing the elimination rates after 2 h, tedizolid phosphate and TZD showed lower elimination rates of less than 5% compared with LZD and VCM (Fig. 5).

The CL_CHDF_ with and without BSA are summarised in Table 2 for each kind of filter membrane. The CL_CHDF_ of tedizolid phosphate, TZD, LZD, and VCM in the presence of BSA were calculated to be 0.12 ± 0.05 L/hr, 0.10 ± 0.03 L/hr, 1.41 ± 0.29 L/hr, and 1.72 ± 0.03 L/hr for the PMMA membrane, and 0.11 ± 0.04 L/hr, 0.17 ± 0.03 L/hr, 1.48 ± 0.06 L/hr, and 1.71 ± 0.13 L/hr for the PS membrane (Table 2). The CL_CHDF_ of tedizolid phosphate, TZD, LZD, and VCM in the absence of BSA were calculated to be 0.73 ± 0.01 L/hr, 0.58 ± 0.11 L/hr, 1.51 ± 0.31 L/hr, and 1.64 ± 0.18 L/hr for the PMMA membrane, and 0.39 ± 0.05 L/hr, 0.59 ± 0.08 L/hr, 1.18 ± 0.13 L/hr, and 1.62 ± 0.17 L/hr for the PS membrane (Table 2). The CL_CHDF_ of tedizolid phosphate and TZD was significantly reduced by the presence of BSA (*P* < 0.01).

**Table 2:** CL_CHDF_ of tedizolid phosphate, TZD, LZD,  and VCM on PMMA and PS membranes

	CL_CHDF_ (L/hr)			
	PMMA		PS	
	BSA 4%	BSA free	BSA 4%	BSA free
tedizolid phosphate	0.12 ± 0.05	0.73 ± 0.01	0.11 ± 0.04	0.39 ± 0.05
TZD	0.10 ± 0.03	0.58 ± 0.11	0.17 ± 0.03	0.59 ± 0.08
LZD	1.41 ± 0.29	1.51 ± 0.31	1.48 ± 0.06	1.18 ± 0.13
VCM	1.72 ± 0.03	1.64 ± 0.18	1.71 ± 0.13	1.62 ± 0.17

## Discussion

Serum albumin concentrations tend to be lower in critically ill patients than in healthy subjects, which may increase the apparent volume of drug distribution and clearance due to high protein-binding rates. In addition, the disposition, metabolism, and excretion of drugs are non-physiological in patients with renal failure. For example, teicoplanin has a high protein-binding rate and is known from previous reports to adsorb on PMMA and PS membranes, affecting clearance [10]. These abnormalities are influenced by many factors, including dialysis status, serum albumin concentration, and concomitant medications. The actual use of drugs in patients with impaired renal function undergoing hemodialysis requires a treatment plan that considers the dosage and timing of administration. Thus, optimizing dosing in critically ill patients is a necessary task; however, there is currently very limited information on substance clearance in CHDF and no guidelines for physicians.

This study compared the affinities of tedizolid phosphate and TZD with those of other anti-methicillin-resistant Staphylococcus aureus agents (LZD and VCM) on two different types of dialysis membranes with different structures, mainly used in CHDF. The PS membrane used in the experiments is the most commonly used membrane in CHDF; PS membranes have an asymmetric structure and are excellent for water removal and filtration. Hypercytokinemia is also observed in septic acute kidney injury, and dialysis membranes with adsorption properties such as PMMA and AN69ST membranes are used. In this study, symmetrically structured PMMA membrane was used as membranes with adsorptive properties, and in vitro experiments were performed to investigate the differences in TZD removal rates in dialysis membranes with different membrane structures.

The PS membrane used in this experiment has an asymmetric structure with a thin active layer on the surface that affects solute permeability and has a high solute elimination capacity from molecular weight substances to β2-microglobulin (β2-MG) with no albumin leakage [15, 16]. Homogeneously structured PMMA membranes made from hydrophobic polymers with an aqueous structure have a high removal capacity for low molecular weight substances. β2-MG is strongly adsorbed from the early stages of processing [15, 16]. Previous reports have shown that the adsorption phenomena of PS and PMMA membranes are different in in vitro experiments using β2-MG solutions, indicating a specificity between the membranes and the solute [17]. The adsorption phenomenon by the membrane starts instantly upon contact between the blood and the membrane; however, the detailed mechanism is still unknown. However, in addition to the factors summarized above, the hydrophobicity, hydrophilicity, surface charge, surface properties of the inner and outer membranes, and the anticoagulant used for treatment are also thought to be important factors in the adsorption of specific solutes by the membrane.

First, experiments were conducted to compare the affinity of the dialysis membrane hollow fibers added to a saline solution to the dialysis membranes. The results showed that TZD, LZD, and VCM were comparable to the control; however, tedizolid phosphate was significantly eliminated compared to the control.

The elimination rates of tedizolid phosphate, TZD, LZD, VCM, filtrate concentration, and CL_CHDF_ were examined using an in vitro CHDF model. The results showed that tedizolid phosphate and TZD showed a slight decrease in concentration compared with LZD and VCM. Although there was a significant difference in the elimination rate of tedizolid phosphate compared with that of the control in the adsorption to the dialysis membrane experiment, the elimination rate was lower in the in vitro CHDF model. This difference may be due to the fact that, in the adsorption experiment, TZD made contact with both the inside and outside of the dialysis membrane, whereas in the in vitro CHDF model, TZD made contact with the inner surface of the membrane only. The filtrate concentrations of LZD and VCM, which showed a significant decrease, were similar to the elimination rates of LZD and VCM. The CL_CHDF_ of VCM in PMMA obtained in this study was close to the value reported previously (1.35 L/hr), which may reflect the in vivo conditions [18]. These results suggested that tedizolid phosphate and its active metabolite, TZD, are less susceptible to CHDF.

However, the present experiments were performed in vitro with dialysis membranes and pseudo-blood (saline containing 4% BSA) and may not fully represent the clinical situation. In addition, other dialysis membranes besides the one used in this study have been used in actual clinical practice. Elucidating these phenomena in a clinical setting would be meaningful, especially in terms of TZD administration and drug selection for hemodialysis patients.

## Conclusion

With the increase in the number of long-term hemodialysis patients and elderly hemodialysis patients, the number of hemodialysis patients requiring antimicrobial agents is increasing; however, because most antimicrobial agents are non-dialyzable, their extracorporeal elimination during hemodialysis treatment is not a major problem. The TZD interview form reported a 10% change in concentration after hemodialysis; however, the dialysis membrane material was unknown.

In this study, we investigated the elimination of the dialysis membranes (PS and PMMA) used in CHDF. In addition, TZD has different pharmacokinetics in oral and intravenous administration. Experiments have proven that TZD is not easily affected by CHDF, providing a new indicator of the actual situation in the medical field.

## Data Availability

The data that support the findings of this study are available from the corresponding author, SY, upon reasonable request.
